# Trait-Based Representation of Biological Nitrification: Model Development, Testing, and Predicted Community Composition

**DOI:** 10.3389/fmicb.2012.00364

**Published:** 2012-10-18

**Authors:** Nicholas J. Bouskill, Jinyun Tang, William J. Riley, Eoin L. Brodie

**Affiliations:** ^1^Ecology Department, Earth Sciences Division, Lawrence Berkeley National LaboratoryBerkeley, CA, USA; ^2^Climate Science Department, Earth Sciences Division, Lawrence Berkeley National LaboratoryBerkeley, CA, USA

**Keywords:** nitrogen cycle, models, biological, geochemistry, mathematical modeling, nitrification

## Abstract

Trait-based microbial models show clear promise as tools to represent the diversity and activity of microorganisms across ecosystem gradients. These models parameterize specific traits that determine the relative fitness of an “organism” in a given environment, and represent the complexity of biological systems across temporal and spatial scales. In this study we introduce a *micro*bial community *trait*-based modeling framework (MicroTrait) focused on *nit*rification (MicroTrait-N) that represents the ammonia-oxidizing bacteria (AOB) and ammonia-oxidizing archaea (AOA) and nitrite-oxidizing bacteria (NOB) using traits related to enzyme kinetics and physiological properties. We used this model to predict nitrifier diversity, ammonia (NH_3_) oxidation rates, and nitrous oxide (N_2_O) production across pH, temperature, and substrate gradients. Predicted nitrifier diversity was predominantly determined by temperature and substrate availability, the latter was strongly influenced by pH. The model predicted that transient N_2_O production rates are maximized by a decoupling of the AOB and NOB communities, resulting in an accumulation and detoxification of nitrite to N_2_O by AOB. However, cumulative N_2_O production (over 6 month simulations) is maximized in a system where the relationship between AOB and NOB is maintained. When the reactions uncouple, the AOB become unstable and biomass declines rapidly, resulting in decreased NH_3_ oxidation and N_2_O production. We evaluated this model against site level chemical datasets from the interior of Alaska and accurately simulated NH_3_ oxidation rates and the relative ratio of AOA:AOB biomass. The predicted community structure and activity indicate (a) parameterization of a small number of traits may be sufficient to broadly characterize nitrifying community structure and (b) changing decadal trends in climate and edaphic conditions could impact nitrification rates in ways that are not captured by extant biogeochemical models.

## Introduction

Understanding the interaction between ecology and biogeochemistry is an important frontier in environmental microbiology. Temporal separation between cellular activity and trace gas flux measurement has hampered efforts to connect, in field studies, the composition, structure, and activity of microbial communities to the biogeochemical processes they catalyze. Given the importance of prokaryotic diversity for ecosystem function (Kassen et al., 2000), a greater understanding of how microbial communities assemble, interact with the changing environment over time is clearly required.

The application of next generation sequencing technology is continually improving our understanding of the spatial and temporal distribution of microorganisms (Caporaso et al., 2012), while metabolomics and proteomics can help contextualize biological interactions with the environment and clarify relationships within and between microbial functional groups (Kujawinski, 2011; Schneider et al., 2012). In contrast, theoretical approaches in microbial ecology have lagged significantly behind these methodological developments (Prosser et al., 2007). Unlike macrofaunal ecology (Webb et al., 2010), mathematical relationships are not routinely applied to explore the implications behind experimental observations. The theoretical background to expand numerical approaches in environmental microbiology could well follow the trait-based approach implemented in models of marine autotrophic phytoplankton (Litchman and Klausmeier, 2008; Follows and Dutkiewicz, 2011). These models have been shown to be valuable tools for understanding how communities assemble (Follows et al., 2007; Litchman et al., 2007), how they change over time (Litchman and Klausmeier, 2006), and the interdependencies between community dynamics and biogeochemistry (Dutkiewicz et al., 2009).

In the current study we expand the trait-based approach to study a critical component of the nitrogen cycle, nitrification. Nitrification, the oxidation of ammonia to nitrite and then nitrate, is a rate-limiting step in the microbially mediated N cycle (Ward, 2008). Nitrification alters the distribution of inorganic N in soil and bridges the input of NH_3_ from N-fixation or organic matter (OM) decomposition to its loss as N_2_O or N_2_ gas via denitrification. In addition, nitrification is closely linked to the carbon cycle as nitrifier activity determines the relative concentration of two major plant and microbial nitrogen sources: ammonia and nitrate. The availability of these two nutrients in turn affects N mineralization rates, soil OM decomposition, denitrification, plant-productivity, and N-loss through leaching or gas efflux.

The initial step of nitrification (NH_3_ → NO_2_) is catalyzed by a phylogenetically restricted group of beta- and gammaproteobacteria (Kowalchuk and Stephen, 2001) and members of the thaumarchaea (Brochier-Armanet et al., 2008). The distribution and abundance of ammonia-oxidizing bacteria (AOB) and ammonia-oxidizing archaea (AOA) in soils and sediments show broad patterns related to substrate (i.e., NH_3_) concentration (Erguder et al., 2009; Wertz et al., 2011), pH (He et al., 2007); (Nicol et al., 2008), OM concentrations (Könneke et al., 2005), dissolved oxygen (Bouskill et al., 2012), and temperature (Avrahami and Bohannan, 2007; Tourna et al., 2008). In addition, while studies of the ecology and biogeochemical importance of the AOA are still nascent, certain ecological trends are evident, such as the ability to nitrify at low pH and grow under oligotrophic substrate concentrations (Erguder et al., 2009; Nicol et al., 2011).

The nitrite-oxidizing bacteria (NOB) belonging to five genera (Nitrobacter, Nitrospira, Nitrococcus, Nitrospina, and Nitrotoga) catalyze the second major step of nitrification (NO_2_ → NO_3_). Few NOB have been isolated from soil and the extent of ecophysiological kinetic data for NOB significantly lags that of AOB. Additionally, PCR primers targeting the functional gene involved in nitrite oxidation (nitrite oxidoreductase) have only recently become available (Vanparys et al., 2007), which has hindered studies of NOB ecology and environmental distribution. Spatial coupling of the two reactions (NH_3_ and NO_2_ oxidation) is well known (Okabe et al., 1999; Schramm et al., 1999) and reduces the likelihood that toxic NO_2_ will accumulate in soils. However, these two oxidative processes can, and often do, become spatially or temporally uncoupled by fluctuating redox or low NO_2_ concentrations selecting against NOB activity, resulting in NO_2_ accumulation. In the following section, we briefly introduce the concept of disaggregating microbial functional groups by specific traits and discuss previous attempts to apply these ideas to microbial ecosystems.

### Trait-based microbial models

Ecosystem activity is closely aligned to the structure and function of endemic microbial communities. These communities catalyze the bulk of biogeochemical reactions related to OM decomposition and nutrient transformations. Although the majority of ecosystem models acknowledge the contribution of prokaryotes in determining the rate of C and N cycling, these models have mainly focused their mechanistic representation on the role physical processes play in regulating biogeochemical cycles. Microbial transformations are often implicitly represented (e.g., Manzoni and Porporato, 2009, and references therein; Parton et al., 1987; Jenkinson and Coleman, 2008) using a specified turnover time for various pools of soil OM (e.g., slow, intermediate, and fast turnover pools). To our knowledge, no modeling frameworks applied at regional or larger scales attempt to represent how the dynamic nature of microbial diversity and activity affects biogeochemical cycling of C, N, or other compounds.

A deterrent to the explicit representation of microbial community dynamics is a lack of understanding of how microbial communities assemble and respond to changing environmental conditions. Microbial communities are extraordinarily diverse, with thousands of different taxa seemingly inhabiting the same environment (Gans et al., 2005; Delong et al., 2006). This diversity can be attributed to a small subset of microorganisms being selected for by the prevailing environmental conditions (Hutchinson, 1961). Selection can be due to a combination of genomic and physiological traits that elevate the fitness of some organisms over their competitors. Therefore, functional diversity is a transient ecosystem property, and as environmental conditions change over time so can microbially mediated reaction rates (e.g., Carney et al., 2007). These changes can have important implications for ecosystem model structure and parameterization.

Trait-based modeling approaches have been reviewed elsewhere (McGill et al., 2006; Green et al., 2008; Webb et al., 2010) and previously applied in ecology (Laughlin, 2011). In microbiology, these models have been used to depict communities of functionally important groups (Allison, 2012) and address questions that field and laboratory experiments are unable to sufficiently answer (Monteiro et al., 2011). These trait-based approaches have attempted to numerically characterize key physiological parameters that contribute toward an ecological strategy.

Nitrifiers are ideal candidates for building and refining trait-based models. They are autotrophic with a simple metabolism largely defined by central physiological processes, such as substrate acquisition (NH_3_ and NO_2_) and substrate use efficiency (number of moles of substrate required to fix one mole of CO_2_). Several decades of ecophysiological studies using different nitrifiers have produced a wealth of data that can be used to mathematically characterize different nitrifier guilds. While heterotrophic organisms can also carry out nitrification (Schimel et al., 1984), at the present time, too little is understood about the distribution, importance and physiology of these organisms (De Boer and Kowalchuk, 2001). Therefore, in this manuscript we describe the development of a *micro*bial community *trait*-based modeling framework (MicroTrait) to simulate the physiology and ecology of autotrophic *nit*rifiers (MicroTrait-N), including an explicit representation of the rates of NH_3_ and NO_2_ oxidation, N_2_O production, and nitrogen pool transformations. We apply MicroTrait-N to examine predicted patterns in nitrifier community diversity and activity across several geochemical gradients.

## Materials and Methods

### Emergent community ecosystem model description (MicroTrait-N)

**Figure 1:**
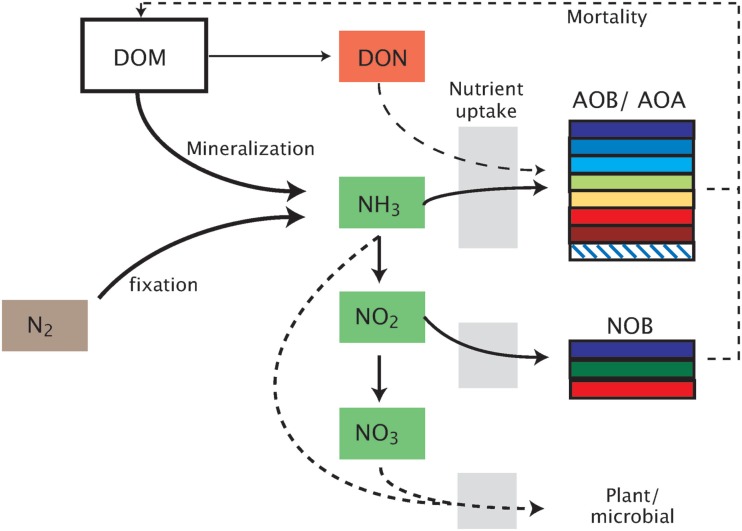
**Schematic representation of the model**. Model abbreviations. DOM, dissolved organic matter; DON, dissolved organic nitrogen; AOB/AOA, ammonia-oxidizing bacteria/archaea; NOB, nitrite-oxidizing bacteria.

MicroTrait-N resolves intra-functional group diversity of the nitrifier populations (AOB, AOA, NOB) by parameterizing multiple guilds spanning a range in the trait-space (Figure 1). Although this nitrifier model will be integrated in an ecosystem model that allows for a wide range of interactions (Tang et al., submitted), we focus here on resolving nitrifier diversity in a competitive environment across a range of conditions, including pH, O_2_, substrate type (NH_3_ or urea), and temperature. Our approach is general enough that it can be applied to nitrifier populations in freshwater and aquatic environments and flexible enough to be used within soil pores. The model is written in Matlab (Matlab R2011b, Natick, MA, USA).

Our guild approach simulates seven lineages of Betaproteobacterial AOB as individual guilds, three NOB guilds, and one AOA guild. The smaller number of NOB and AOA guilds reflects the lack of relevant ecophysiological studies of these groups. Intra-guild diversity is parameterized by allowing a range of values for each trait (Table 1), based on previous ecophysiology studies (Loveless and Painter, 1968; Suzuki, 1974; Suzuki et al., 1974; Drozd, 1976; Belser, 1979; Belser and Schmidt, 1979; Glover, 1985; Keen and Prosser, 1987; Prosser, 1989; Nishio and Fujimoto, 1990; Verhagen and Laanbroek, 1991; Laanbroek and Gerards, 1993; Jiang and Bakken, 1999; Schramm et al., 1999; Gieseke et al., 2001; Koops and Pommerening Röser, 2001; Cébron et al., 2003; Martens-Habbena et al., 2009; Schreiber et al., 2009). Further information concerning the derivation of trait values is given in the supplemental material. Given the paucity of within-guild information, we assumed a uniform probability density of trait values across each trait range. We can increase the number of guilds as more information becomes available to distinguish intra-guild diversity. We performed several types of simulations investigating the role of pH, temperature, decoupling nitrite, and ammonia oxidation, and pulsed NH_3_ inputs, by: (1) using the mean value of each trait; (2) performing Monte Carlo (MC) simulations to account for intra-guild diversity; and (3) running the model in equilibrium and dynamic steady state cycle modes to characterize the impact of temporal forcing variation on predicted emergent microbial community structure.

**Table 1:** **Trait values across the different guilds**.

GUILD	DON	$V_{\max}^{\text{N}{\text{H}}_{3}}\left(\text{da}{\text{y}}^{-1}\right)$	$K_{M}^{\text{N}{\text{H}}_{3}}\left(\mu \text{M}\right)$	μmax (day^−1^)	$K_{\text{M}}^{{\text{O}}_{2}}\left(\mu \text{M}\right)$	R_CN_	Temperature optimum (K)	Phylogenetic affiliation
AOB(1)	−	0.38–1.1	30–61	0.02–0.09	6.9–17.6	0.04–0.08	290–95	*Nitrosomonas europaea*
AOB(2)	−	0.24	14–43	0.01–0.06	3.6–12.4	0.08–0.09	287–99	*Nitrosomonas communis*
AOB(3)	+	0.4–0.9	19–46	0.04*	4.2–14	0.06*	287–99	*Nitrosomonas nitrosa*
AOB(4) AOB(5)	+	0.4–0.8	1.9–4.2	0.06–0.08	1.4–4.7	0.02–0.05	287–99	*Nitrosomonas oligotropha*
	+	1.0–1.04	50–52	0.018	11–23	0.04–0.07	287–99	*Nitrosomonas marina*
AOB(6)	+	0.8–1.2	42–59	0.04*	11–23	0.02–0.03	275–86	*Nitrosomonas cryotolerans*
AOB(7)	+	0.42–0.9	1.4–11	0.07–0.08	0.7–1.2	0.06	285–99	*Nitrosospira* spp.
AOA	?	0.4–0.8	0.01–0.02	0.09–0.11	0.015	0.05	285–99	*Nitrosopumilus maritimus*
NOB(1)	−	0.8–1.9	4–10	0.3–0.7	40–80	0.01–0.03	285–95	*Nitrospina* spp.
NOB(2)	−	2–3.2	45–260	0.8–1.0	60–120	0.04–0.07	275–302	*Nitrobacter* spp.
NOB(3)	−	0.4–4	24–120	0.5–0.7	35–70	0.03–0.06	273–84	*–*

Column headers represent the following; DON, ability to use dissolved organic nitrogen (“?” indicates the ability to use DON is unknown. In this case the guild is assumed to be unable to use DON); $V_{\text{MAX}}^{\text{N}{\text{H}}_{\text{3}}},$ maximal substrate uptake rate; $K_{\text{M}}^{\text{N}{\text{H}}_{\text{3}}},$ half saturation constant for NH_3_; μ_MAX_, maximum growth rate; $K_{\text{M}}^{{\text{O}}_{\text{2}}},$ half saturation constant for O_2_, R_CN_, substrate use efficiency, ratio of NH_3_ moles required to fix one mole of CO_2_, *indicates this value has not been measured and it’s derivation is based on an average across the values for different guilds.

### Representing autotrophy

In the model, the biomass of each nitrifier guild is represented with five variables: (1) total cell biomass (denoted *B*_T_, which may represent the ammonia-oxidizing organism (AOO, i.e., AOB + AOA) as *B*_TA_ or the NOB, *B*_TN_); (2) carbon biomass (*B*_C_); (3) nitrogen biomass (*B*_N_); (4) Cellular quotas for carbon (*Q*_C_); and (5) cellular quotas for nitrogen (*Q*_N_). The latter two are defined relative to total biomass (i.e., *Q*_C_ = *B*_C_/*B*_T_; *Q*_N_ = *B*_N_/*B*_T_). Carbon biomass increases by fixing CO_2_ through the ribulose-bisphosphate enzyme using energy produced during the oxidation of either NH_3_ or NO_2_ (Figure 1). Cell division of the AOO and NOB is governed by Droop kinetics (Droop, 1973):

(1) $$d_{B,j}^{i}=\max\left(1-\frac{\overset{\min}{\underset{B\text{,}j}{Q}}}{\overset{i}{\underset{B,j}{Q}}},0\right)$$

where $Q_{B,j}^{i}$ represents the biomass quota (i.e., *Q*_C_ or *Q*_N_) of the *i*th guild for the *j*th element. Here *j* represents either C or N. The minimum quota for carbon is 1 and for nitrogen is 1/13.2 (according to the Redfield Ratio). The carbon and nitrogen constraints are then applied to regulate the cell division rate (*D*_B_) with Liebig’s law of the minimum (van der Ploeg, 1999):

(2) $$D_{B}=\mu_{\text{max}}^{B}\min\left\{d_{\text{i}}\right\}B_{\text{T}}$$

where $\mu_{\max}^{B}\left(d^{-1}\right)$ is the nitrifier maximum specific growth rate (Table 1). Ammonia oxidation in AOO is modeled with Briggs–Haldane kinetics (Koper et al., 2010):

(3) $$V_{AOB}^{\text{N}{\text{H}}_{3}}=V_{\max}^{\text{N}{\text{H}}_{3}}\frac{\left[\text{N}{\text{H}}_{3}\right]}{K_{M}^{\text{N}{\text{H}}_{3}}+\left[\text{N}{\text{H}}_{3}\right]\left(\frac{1+\left[\text{N}{\text{H}}_{3}\right]}{K_{\text{i}}^{\text{N}{\text{H}}_{\text{3}}}}\right)}\frac{\left[O_{2}\right]}{K_{M}^{O_{2}}+\left[O_{2}\right]}B_{\text{TA}}$$

Here, $V_{\max}^{\text{N}{\text{H}}_{3}}\left(\text{M}{\text{S}}^{\text{ - 1}}\right)$ is the maximum substrate (NH_3_) uptake rate, *K*_M_ is the half saturation constant for NH_3_ or O_2_ (μM; Table 1), and $K_{i}^{\text{N}{\text{H}}_{\text{3}}}$ is the NH_3_ inhibition constant for AOB (μM; Table 1). Substrate concentrations are in M (mol L^−1^). CO_2_ uptake follows Michaelis–Menten kinetics:

(4) $$V_{AOB}^{\text{C}{\text{O}}_{2}}=V_{\max}^{\text{C}{\text{O}}_{2}}\frac{\left[\text{C}{\text{O}}_{2}\right]}{K_{m}^{\text{C}{\text{O}}_{2}}+\left[\text{C}{\text{O}}_{2}\right]}$$

where $V_{\max}^{\text{C}{\text{O}}_{2}}$ is guild-specific and depends on energy yielded by ammonia oxidation and the efficiency of CO_2_ fixed relative to NH_3_ oxidized:

(5) $$V_{\max}^{\text{C}{\text{O}}_{2}}=\frac{Y_{\text{N}}^{\text{C}{\text{O}}_{\text{2}}}V_{\max}^{\text{N}{\text{H}}_{3}}}{Q_{\text{N}}}\max\left(1-\frac{r_{\text{CN}}-\overset{\min}{\underset{\text{CN}}{r}}}{\overset{\max}{\underset{\text{CN}}{r}}-r_{\text{CN}}^{\min}},0\right)$$

where $Y_{\text{N}}^{\text{C}{\text{O}}_{\text{2}}}$ (unitless) is the guild-specific substrate use efficiency (number of moles of NH_3_ oxidized per mole of CO_2_ fixed, Table 1) and represents the C:N ratio (i.e., the Redfield ratio; Redfield, 1958) of each nitrifier guild and $r_{\text{CN}}^{\min}=6.6$ and $r_{\text{CN}}^{\max}=13.2$, which are use to reflect the autotrophic nature of the nitrifiers.

Growth of the *i*th AOB biomass over time is calculated as:

(6) $$\frac{dB_{\text{TA}}^{i}}{dt}=\mu_{\text{max}}^{i}\min\left\{d_{i}\right\}\overset{i}{\underset{\text{TA}}{\text{B}}}-\Delta B_{\text{TA}}^{\text{i}}-\frac{1}{4}\left(D_{\text{A}}^{\text{N}{\text{O}}_{2}}+D_{\text{A}}^{\text{NO}}\right)$$

Here, Δ (s^−1^) is the first order microbial mortality rate and *D*_A_ is biomass loss (M s^−1^) attributable to the detoxification of NO_2_ following the uncoupling of AOB and NOB mediated reactions (see below). Total biomass loss is the sum of that required to convert NO_2_ → NO and NO → N_2_O, and the 1/4 represents the stoichiometric relationship between biomass and NO_2_ detoxification (i.e., 4NO_2_ + CH_2_O → 4NO + CO_2_ + 3H_2_O; 8NO + 2CH_2_O → 4N_2_O + 2CO_2_ + 2H_2_O).

The NOB gains energy to fix CO_2_ to biomass via the oxidation of NO_2_ → NO_3_. NO_2_ uptake rate is modeled by:

(7) $$V_{\text{NOB}}^{\text{N}{\text{O}}_{2}}=V_{\text{max}}^{\text{N}{\text{O}}_{2}}\frac{\left[\text{N}{\text{O}}_{2}\right]}{K_{M}^{\text{N}{\text{O}}_{2}}+\left[\text{N}{\text{O}}_{2}\right]}\frac{\left[{\text{O}}_{2}\right]}{K_{M}^{{\text{O}}_{2}}+\left[{\text{O}}_{2}\right]}B_{\text{TN}}$$

where the different terms in Eq. 7 are analogous to those in Eq. 3. The uptake of CO_2_ occurs via the same pathway as for AOO (Eqs 4 and 5) and the biomass of the *i*th NOB guild varies as:

(8) $$\frac{dB_{\text{TN}}^{\text{i}}}{dt}=\mu_{\max}^{i}\min\left\{d_{\text{i}}\right\}\overset{i}{\underset{\text{TN}}{B}}-\Delta B_{\text{TN}}^{i}$$

### Nitrous oxide production

**Figure A1:**
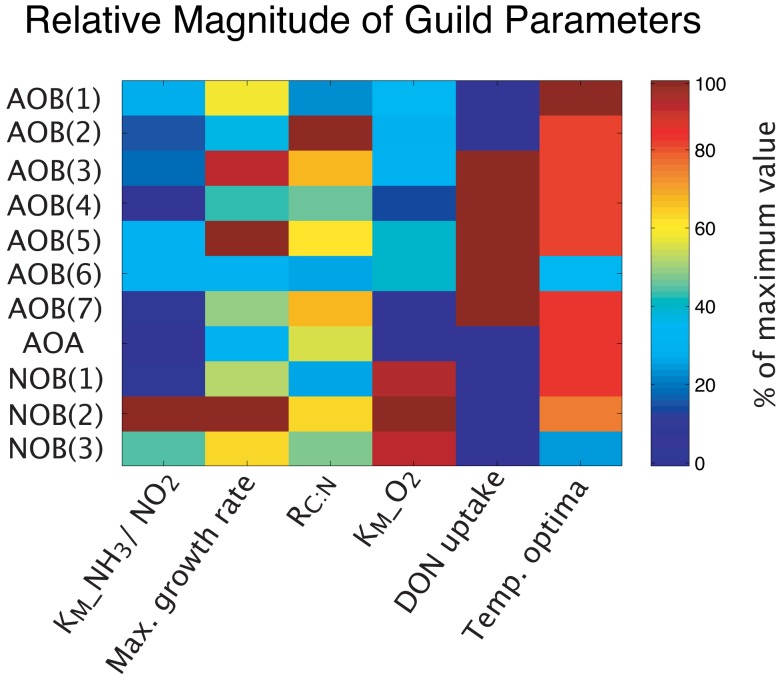
**Relative magnitude of guild parameters**.

N_2_O is produced by AOO via two distinct pathways: (1) decomposition of the hydroxylamine intermediate and (2) the likely more significant mechanism of NO_2_ detoxification (Figure A1 in Appendix; Frame and Casciotti, 2010; Kool et al., 2011; Stein and Klotz, 2011). Under the first pathway, N_2_O production is modeled as a linearly related fraction of hydroxylamine decomposition (Frame and Casciotti, 2010). The second pathway simulates the detoxification of accumulated NO_2_ as the two steps of nitrification become uncoupled. This decoupling can occur because NOB have a lower affinity for O_2_ than the AOB; therefore as O_2_ is consumed during nitrification (or in low O_2_ environments), the two reactions may become spatially or temporally uncoupled. NO_2_ toxicity stimulates a detoxification pathway converting NO_2_ to N_2_O via NO. This detoxification pathway is potentially the more significant mechanism by which AOB produce N_2_O. AOA have recently been shown to produce N_2_O (Santoro et al., 2011), although the mechanism has not yet been elucidated. Therefore, in the present version of the model we predict AOA N_2_O production using the same relationships as for AOB.

As NO_2_ concentrations become toxic to AOO, their growth and NH_3_ uptake decline. We represent these transitions by modifying an organism’s affinity for NH_3_ as a function of NO_2_, NO, and O_2_ concentrations:

(9) $$K_{\text{M}}^{\text{N}{\text{H}}_{\text{3}}}=K_{\text{Mb}}^{\text{N}{\text{H}}_{3}}\left[1+K_{\text{d}}^{\text{max}}\frac{\left[\text{C}\right]}{\left[{\text{O}}_{2}\right]}\right]$$

where $K_{\text{Mb}}^{\text{N}{\text{H}}_{\text{3}}}$ is the base NH_3_ affinity, $K_{\text{d}}^{\text{max}}$ is the affinity constant for NO_2_ or NO during detoxification, and [C] represents the concentration (M) of either NO_2_ or NO. Energy for detoxification is assumed to come from the degradation of microbial biomass resulting in the output of CO_2_.

### Nutrient pool transformations

The dynamic aqueous NH_3_ concentration ([NH_3_] (M) depends on a balance between losses from oxidation $\left(V_{\text{N}{\text{H}}_{3}}^{\text{E}}\right),$uptake into biomass of AOO $\left(V_{\text{N}{\text{H}}_{\text{3}}}^{\text{B}}\right),$ and NOB $\left(V_{\text{N}{\text{H}}_{3}}^{\text{NOB}}\right),$ and inputs resulting from biomass breakdown during detoxification summed across the total number of AOO guilds (*n*_A_) and NOB guilds (*n*_N_):

$$\begin{array}{llll} \frac{d\left[\text{N}{\text{H}}_{3}\right]}{dt} & =-\sum_{i=1}^{i=n_{\text{A}}}\left(V_{\text{N}{\text{H}}_{\text{3}}}^{\text{E}}+V_{\text{N}{\text{H}}_{3}}^{\text{B}}\right)-\sum_{I=1}^{i=n_{\text{N}}}V_{\text{N}{\text{H}}_{3}}^{\text{NOB}} & & \\ & \;+\frac{1}{4}\sum_{i=1}^{i=n_{\text{A}}}\left(D_{\text{A}}^{\text{N}{\text{O}}_{2}}+D_{\text{A}}^{\text{NO}}\right) & & \text{(10)} \end{array}$$

where the 1/4 represents the stoichiometry of the detoxification reaction using biomass for energy. The dynamic NO_2_ concentration depends on uptake by NOB to generate energy and losses via detoxification by AOB:

(11) $$\frac{d\left[\text{N}{\text{O}}_{2}\right]}{dt}=\sum_{i\text{ = 1}}^{i\text{ = }n_{\text{A}}}V_{\text{N}{\text{H}}_{\text{3}}}^{\text{E}}-\sum_{i\text{ = 1}}^{i\text{ = }n_{\text{N}}}V_{\text{N}{\text{O}}_{\text{2}}}^{\text{E}}-\sum_{i\text{ = 1}}^{i\text{ = }n_{\text{A}}}D_{\text{A}}^{\text{N}{\text{O}}_{\text{2}}}$$

### Model evaluation

#### Resolution of nitrifier diversity across geochemical gradients

We tested MicroTrait-N by examining how nitrifier diversity varies across geochemical gradients in pH, substrate concentration [i.e., (NH_3)_], and temperature and compared predictions of this diversity against published studies. Accuracy of modeled communities was gaged by relating the steady state modeled nitrifier diversity to its likely phylogeny based on literature sources of the derived trait values. In addition, an evenness statistic (*J^i^*) is ascribed to each community;

$$J^{i}=\overset{\text{S}}{\underset{i\text{ - 1}}{\sum }}\frac{\left[\left(p_{i}\right)\ln\left(p_{i}\right)\right]}{\ln\left(\text{S}\right)}$$

where represents the relative proportion of the *i*th species, and S is the species richness (Mulder et al., 2008). The evenness statistic varies between 0 and 1, with 1 indicating an equal contribution of each guild to the total biomass. The model also predicts rates of NH_3_ oxidation and N_2_O production that we report as 30 days running averages.

#### Physicochemical impacts on nitrifier diversity and activity

We applied a step-wise approach to analyze the impacts of geochemical variables, temporal dynamics of substrate inputs, and combinations of these variables on nitrifier diversity and activity. The five groups of modeling scenarios include sensitivity analyses of the impacts of (i) pH; (ii) temperature; (iii) decoupling during NO_2_ detoxification; and (iv) dynamic substrate inputs. For the fifth modeling scenario, (v) we computed predicted community structure with a limited set of available observations.

##### pH impacts

pH is a determinant of nitrifier diversity, in part, due to its regulation of NH_3_ concentrations. The NH_4_:NH_3_ ratio increases as pH decreases (Li et al., 2012), possibly selecting for nitrifiers adapted to low substrate concentrations. We performed model simulations across pH gradients spanning neutral to slightly acidic conditions (7.8–4.5). For each guild, the model was run with an integration time of 6 months, which allowed the community biomass to come to a steady state. Simulations were initialized with 1 × 10^−5^ M NH_3_ and non-limiting concentrations of O_2_ and CO_2_ (both 1 M × 10^−3^ M). Two further substrate pulses (of 1 × 10^−6^ NH_3_) following 2 and 4 months were necessary to prevent the communities becoming substrate limited and maintain them at steady state.

##### Temperature impacts

Temperature has also been shown to play an important role in determining the diversity of ammonia-oxidizing communities in terrestrial and aquatic ecosystems (Erguder et al., 2009; Prosser, 2011). We applied in the model a temperature-activity relationship based on previously published data (Ratkowsky et al., 2005; Follows et al., 2007) that accounts for a different temperature optima across the guilds (Table 1). We simulated a temperature range of 5 to 30°C in 5°C increments under initial conditions of NH_3_ = 5 × 10^−5^ M and pH = 7.8.

##### Decoupling nitrification reactions

We simulated the forced reduction of NO_2_ to N_2_O during AOO detoxification by initializing the model to steady state over 6 months under initial conditions of 1 × 10^−5^ M NH_3_, pH = 7.8 and temperature = 20°C. At steady state, the NOB activity was turned off and then simulations were run for a further 6 months. A simultaneous control experiment extended the steady state for a further 6 months maintaining NOB activity.

##### Pulsed substrate inputs

NH_3_ availability is considered to be a major determinant of AOO diversity (Bouskill et al., 2011; Prosser, 2011) and the rate of N_2_O efflux (Elberling et al., 2010). Nitrifiers show wide physiological breadth with respect to enzyme kinetics (*V*_max_ and *K*_m_) and different communities dominate based on the magnitude of substrate inputs (Mahmood et al., 2006). We tested the impact of NH_3_ availability by simulating community diversity and activity in response to pulsed NH_3_ input events. Under a constant pH (7.8) and temperature (25°C), NH_3_ was initially input at a concentration of 1 × 10^−6^ M and increased on 2-month cycles to 5 × 10^−5^ M.

##### Comparisons with observed data

We tested the baseline MicroTrait-N predictions by comparing against published data from five Alaskan ecosystems (Petersen et al., 2012). That dataset combines nitrification rate measurements with a quantification of the different nitrifier groups (AOB and AOA) facilitating a direct comparison with the output of our model. Petersen et al. (2012) also report a comprehensive list of chemical data, which satisfy the input requirements of the simulation’s initial conditions. Furthermore, in contrast to our earlier simulations evaluating community composition at a fixed substrate concentration and low pH (down to 4.5), this dataset represents low pH soils (4.8–4.3) with high substrate concentrations. For these simulations initial conditions are given in Table A1 in Appendix with temperature = 15°C and simulations were run for 6 months. The model was initialized with mean trait values and then simulations were replicated using the MC approach and five analogs per guild (with each analog representing a stochastically chosen set of trait values across the uniform probability distribution. For comparison, data from two of the sites are replicated using an MC code with a normal distribution. Using the normalized distribution of traits produces little effect on the model output. See appendix).

## Results

### Physicochemical impacts on nitrifier diversity and activity

In this subsection we describe results from our modeling scenarios and comparison of predicted data with observations.

#### pH impacts

**Figure 2:**
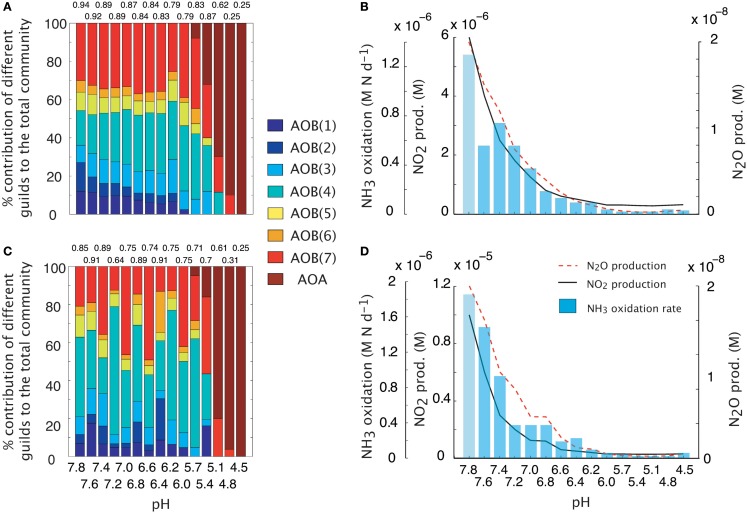
**Simulations of AOO diversity and activity across a pH gradient**. Community evenness values are given above the stacked bars. **(A)** Community diversity (proportion of total biomass) predictions using mean trait values. **(B)** Simulated nitrifier activity (NH_3_ oxidation, NO_2_ production, N_2_O production) using mean trait values. **(C)** Community diversity (proportion of total biomass) predictions using Monte Carlo simulations of multiple AOO analogs (*n* = 5 analogs per guild). **(D)** Simulated nitrifier activity (NH_3_ oxidation, NO_2_ production, N_2_O production) using Monte Carlo simulations of multiple AOB analogs (*n* = 5 analogs per guild).

We simulated a pH gradient from approximately neutral (pH = 7.8) to acidic (pH = 4.5) conditions and recorded diversity and activity (NH_3_ oxidation rate and N_2_O production). During the hydrolysis reaction of NH_3_, the ratio NH_4_:NH_3_ increased hyperbolically as pH decreased. Thus, at pH < 5, the extremely low [NH_3_] encouraged the growth of oligotrophic ammonia oxidizers. Both baseline (i.e., fixed trait values, Figures 2A,B) and MC (Figures 2C,D) approaches showed a decline in AOB community evenness with decreasing pH. The highest evenness values are predicted around neutral values where AOB guilds 7 [AOB(7)] and 4 [AOB(4)] dominate. As pH decreases, community diversity declines until the AOA guild dominates. Although both simulations had similar trends in diversity, the multiple analog experiments (Figures 2C,D) predicted more variability in community diversity, as evidenced by more variable evenness values. Predicted nitrifier activity (as indicated by NH_3_ oxidation rates and N_2_O production) also declined with decreasing pH from a maximum NH_3_ oxidation rate of 1.9 M N day^−1^ to less than 0.1 M N day^−1^. Predicted N_2_O production was linearly related to NH_3_ oxidation (data not shown, *r* = 0.98, *p* = 0.001, slope = 0.94) indicating the AOB and NOB reactions were coupled regardless of the pH and N_2_O was primarily by hydroxylamine decomposition.

#### Temperature impacts

**Figure 3:**
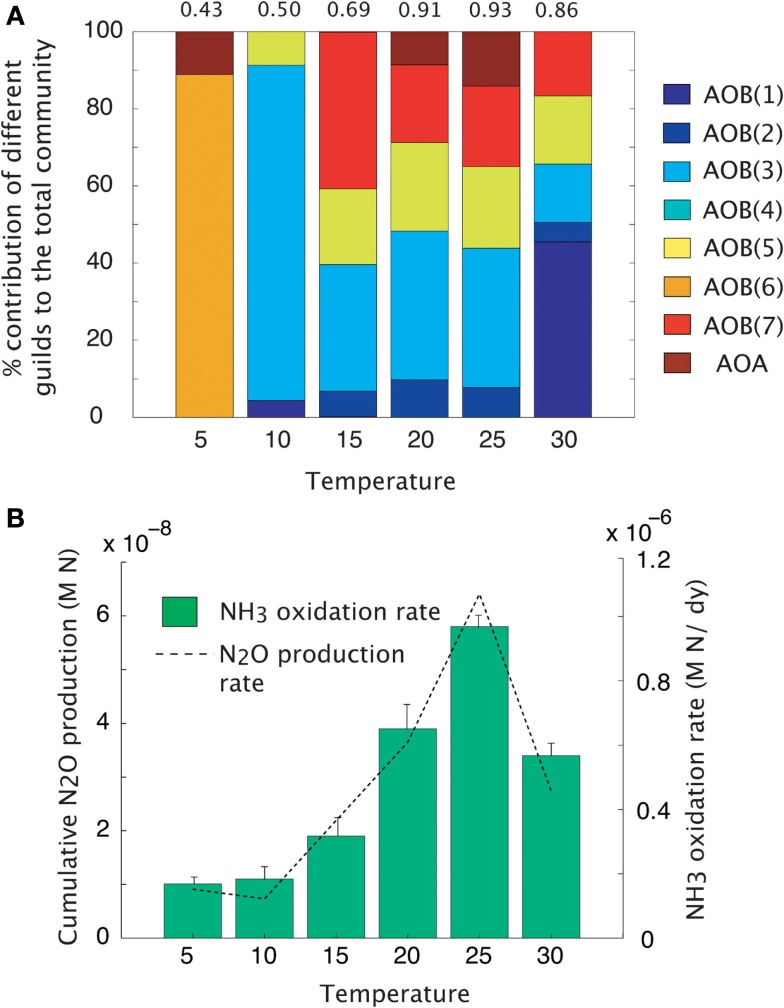
**Mean trait-value AOO community diversity and activity across a temperature gradient**. **(A)** Stacked bar chart depicts community diversity as a proportional contribution to the total community biomass. The evenness value is given above the plot. **(B)** Rates of NH_3_ oxidation (bar chart) and gross N_2_O production (line graph). Error bars are the result of multiple simulations (*n* = 3).

Maximal rates of ammonia oxidation were simulated at 25°C (Figure 3B). Maximal oxidation rates coincided with the highest community evenness. At low temperature, AOO communities were dominated by the cold-adapted AOB(6) guild (Table 1, Figure 3A), which represents *Nitrosmonas cryotolerans*. The AOA guild was also important at this temperature (Figure 3A). With increasing temperatures up to 25°C, the AOB(3) and AOB(7) guilds became more competitive and began to dominate the community. When the temperature reached 30°C, the AOB(1) guild dominated. N_2_O production mirrored that of NH_3_ oxidation indicating that N_2_O production resulted from hydroxylamine decomposition under these conditions.

#### Decoupling nitrification reactions

**Figure 4:**
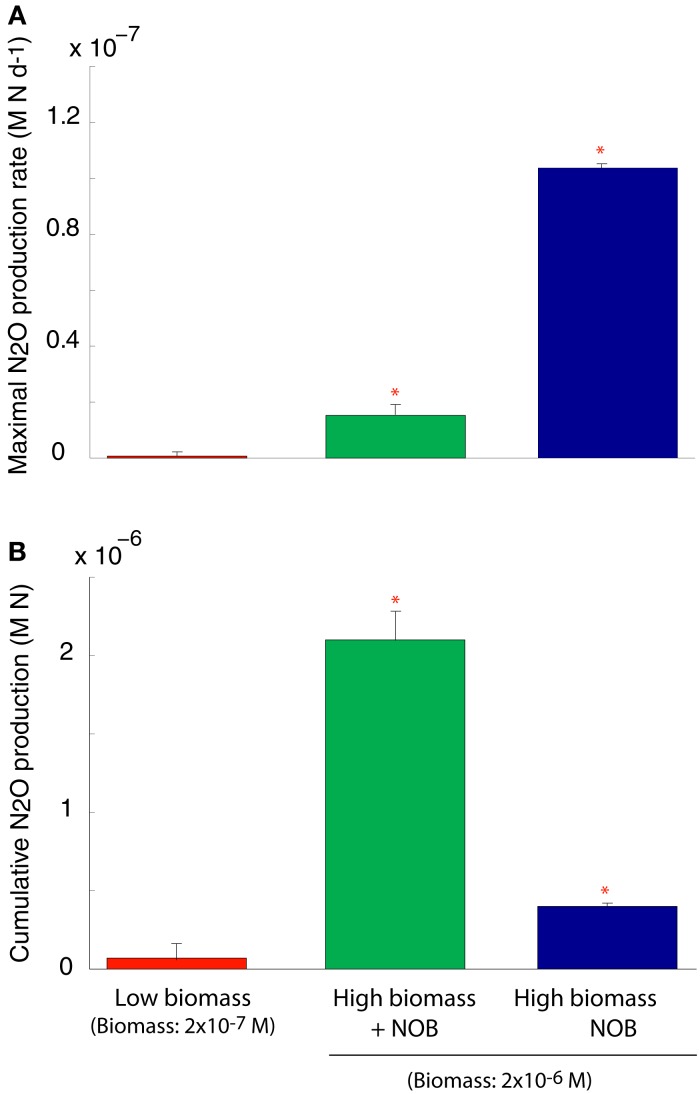
**N_2_O production under a coupled AOB-NOB nitrification reaction and also as the AOB-NOB reaction becomes uncoupled and the detoxification reaction is activated**. **(A)** Maximal rate of N_2_O production **(B)** Cumulative N_2_O production over the 6-month simulation. Error bars are the result of three simulations per temperature.

We simulated N_2_O production through two pathways described above (Figure A1 in Appendix). After running the simulations to steady state biomass, the NOB were removed allowing rapid accumulation of NO_2_ and invoking a detoxification response in the AOO. NO_2_ was rapidly converted to N_2_O, via NO, using cellular biomass as an energy source. This conversion resulted in a transient N_2_O production rate significantly higher than in the scenarios with a steady state community and when the NOB were present (ANOVA, *p* < 0.05; Figure 4A). Despite a higher N_2_O production rate in the absence of NOB, cumulative production of N_2_O over 6 months was significantly (ANOVA, *p* < 0.05) lower than when NOB were present (Figure 4B) due to the creation of an unstable half reaction (lacking NO_2_ oxidation) resulting in a rapid crash in AOO community biomass (data not shown).

#### Pulsed substrate input

**Figure 5:**
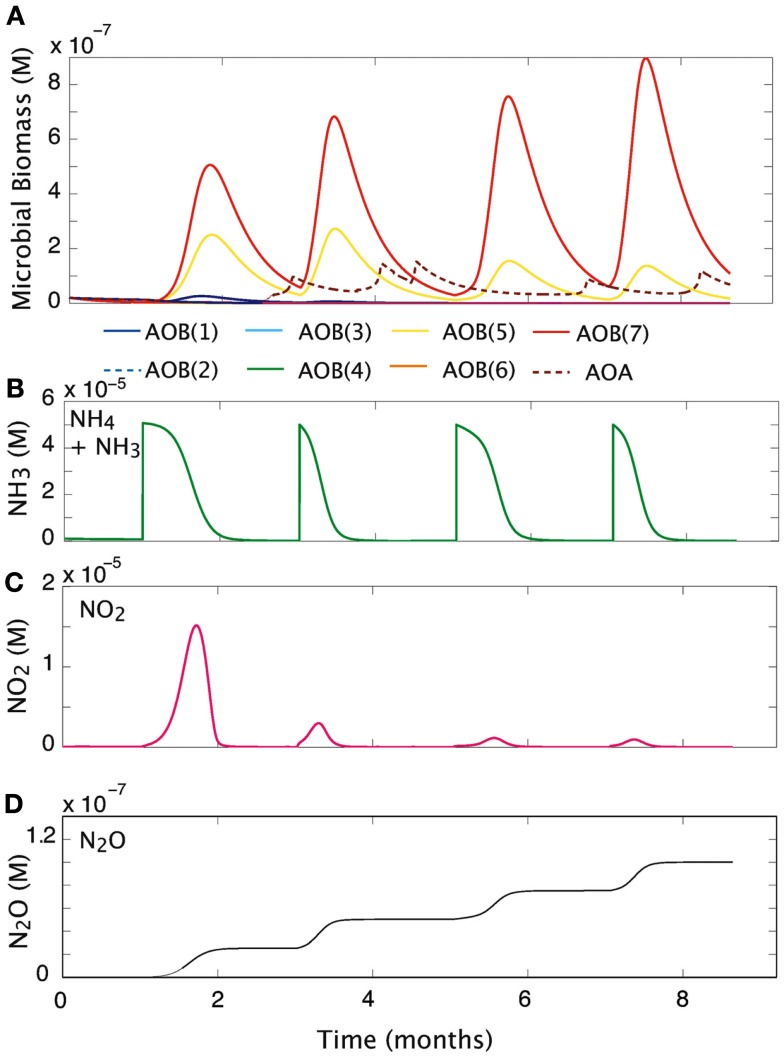
**Community response to pulsed substrate input**. **(A)** Changes in AOO biomass over time. **(B)** Substrate concentration (M). **(C)** Nitrite dynamics over time. **(D)** Production of N_2_O over time.

We simulated the response of our imposed simple community (seven AOB guilds; one AOA guild; and three NOB guilds) to pulsed input of substrate over a 9-month period (Figure 5). Over time, and with evenly spaced pulsed events, the evenness of the community declines slightly from 0.76 to 0.58 as one guild, AOB(7), begins to dominate. Pulses of NH_3_ are drawn down more quickly as the biomass of AOB increases. However, the second pulse of NH_3_ results in its most rapid drawdown due to a high cumulative biomass and greater diversity of AOO (Figures 5A,B). As NOB biomass increases, NO_2_ demand increases, and the NO_2_ is oxidized as rapidly as it is produced (Figure 5C). In the present simulation we did not allow for diffusion, and this resulted in an accumulation of N_2_O (Figure 5D), nevertheless, the rate at which it is produced reflects the pulses of NH_3_ into the system. The initial pulse elevates NH_3_ concentrations from 1 × 10^−7^ to 5 × 10^−6^ and results in a five-fold increase in the biomass of AOB(7), a four-fold increase in AOB(5), and a small response in AOB(1). As NH_3_ is drawn down to lower concentrations (<1 × 10^−6^ M) AOA briefly become the dominant nitrifiers. While AOA biomass peak when substrate concentrations are low, they are inhibited by subsequent substrate pulses.

#### Comparison with environmental data

**Figure 6:**
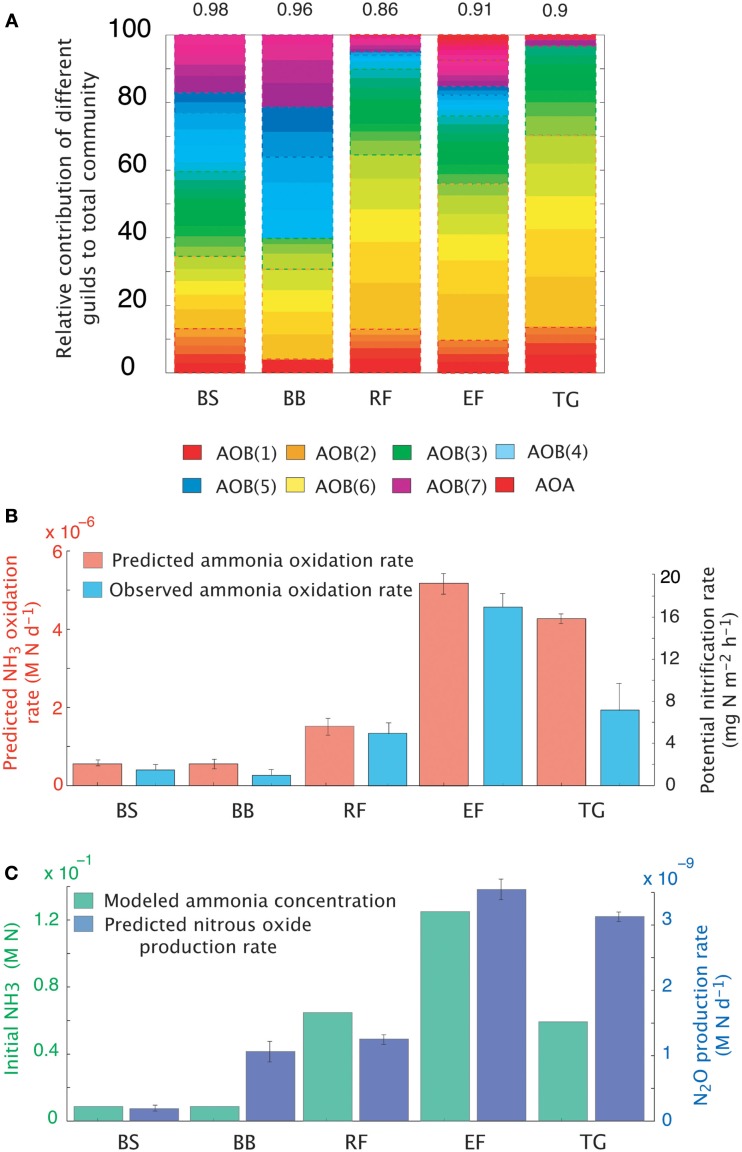
**Simulations of the activity and diversity of AOB communities in high-latitude ecosystems**. **(A)** Monte Carlo simulations of multiple AOB analogs (*n* = 5 analogs per guild) across the different sites. Each guild is represented by a distinct color. Subtle differences in the shade of that color demarcate the different analogs/guild. A box outlines the boundaries of each guild’s biomass. Evenness statistic given above the bar plots. **(B)** NH_3_ oxidation rates from just simulated and observed data. **(C)** Predicted rates of N_2_O production and measured NH_3_ concentrations. Error bars are the result of multiple simulations (*n* = 3). BS, Black Spruce; BB, Bog Birch; RF, Rich Fen; EF, Emergent Fen; TG, Tussock Grassland.

The dataset presented by Petersen et al. (2012) examined AOO community diversity across five-plant community types characteristic of the interior of Alaska. These soils were characterized by high substrate concentrations (range = 7.3 × 10^−3^ to 0.1 M NH_3_) and low pH (4.3–4.8). These observations therefore provide a comparison to our earlier examination of a pH gradient with a fixed substrate concentration. The model predicted that, in contrast to our previous predictions at low pH and NH_3_ substrate levels (Figure 2), bacteria dominated the AOO community at these sites (Figure 6A). Using mean values for traits, the Black Spruce and Bog Birch sites were dominated by AOB(7) and AOB(3) in the case of the Bog Birch site. The Tussock Grassland, Emergent Fen, and Rich Fen also showed lower evenness and were generally dominated by one guild [AOB(1)] accounting for approximately 90% of the total AOB biomass. The AOA guild was never a significant component of the community diversity under these conditions (data not shown). Within-guild diversity was represented using MC simulations that stochastically assigned traits to multiple analogs of each guild. The community composition that emerged when using this approach was different than when traits were represented by their mean values. For example, the AOA became more prominent in the MC simulations, although they were still only a relatively small proportion (2–4%) of the Fen communities and Tussock grassland (Figure 6A).

Predicted trends in NH_3_ oxidation rates (Figure 6B) correlated with the observed data (Figure 6B; *r* = 0.96, *p* = 0.007). The highest oxidation rates were associated with the highest NH_3_ concentrations at the Emergent Fen site (4.9 × 10^−4^ M N day^−1^) and with the lowest rates at the Black Spruce and Bog Birch sites (9 × 10^−5^ and 9 × 10^−6^ M N day^−1^ respectively). MicroTrait-N predictions of N_2_O production also correlated with NH_3_ concentrations and oxidation rates (Figure 6C), albeit not significantly (*r* = 0.69, *p* = 0.19), and were 85 times higher at the Emergent Fen site (3.6 × 10^−6^ M N day^−1^) than the Black Spruce (4.3 × 10^−8^ M N day^−1^).

## Discussion

Oxidation of NH_3_ to NO_3_ is an important process that couples N-inputs and losses via denitrification and influences the availability of N in terrestrial and marine environments (Ward, 2008; Prosser, 2011) with important implications for carbon cycling (Doney et al., 2007). A better understanding of the ecological factors that determine the activity and diversity of the chemoautotrophic nitrifiers will therefore improve our understanding of N-transformations and N-emissions. To that end we describe here a model simulating nitrifier community development as a function of environmental conditions, allowing both community diversity and the rate of nitrification to change across environmental gradients.

### Guild characterization

MicroTrait-N simulates nitrifier diversity using a guild model loosely based on phylogenetic affiliations (Koops and Pommerening Röser, 2001), with differences in key ecophysiological characteristics (e.g., DON usage, *K*_M_ values). Several of the results across gradients showed plausible representation of the dominant nitrifiers guilds emerging on the basis of environmental conditions (discussed below). Our guild characterization recognizes several guilds of the *Nitrosomonas* [AOB(1-6)], one guild of the *Nitrosospira* [AOB(7)] and the AOA, and three guilds of the NOB. The guilds resolve broadly into oligotrophic and copiotrophic groups (Kassen et al., 2000; Lauro et al., 2009). For example, the AOB(5) and AOB(7) guilds have copiotrophic-like characteristics, responding rapidly to substrate pulses (Figure 5A), while the AOA guild is only competitive as substrate is either drawn down to concentrations ≤1 μM (Figure 5A) or when pH reduces NH_3_ availability (Figure 2).

The MicroTrait-N model structure is currently weighted in favor of guilds with cultured members and likely under-represents the importance of the AOA. The AOA are known to be in high abundance in both oceanic (Bouskill et al., 2012) and terrestrial (Leininger et al., 2006) environments. However, while it is likely that marine AOA are chemoautotrophic organisms and play an important role in marine nitrification, AOA possibly span a more complicated functional space in terrestrial systems. Attempts to draw correlations between the abundance of terrestrial AOA and NH_3_ oxidation rates have produced mixed results (Di et al., 2009); (Jia and Conrad, 2009). In MicroTrait-N, parameterization of AOA kinetics is extrapolated from a few published cultures (Martens-Habbena et al., 2009; Lehtovirta-Morley et al., 2011). The model consequentially represents the AOA as oligotrophs, dominating nitrifying conditions under low NH_3_ concentrations, and becoming outcompeted or possibly inhibited under higher NH_3_. The AOA:AOB relationship provides some support for the idea that AOA are oligotrophic, with ratios increasing as substrate concentrations decrease (Mosier and Francis, 2008; Bouskill et al., 2012), while AOA have generally been reported in low abundance within engineered systems of high NH_3_ concentrations (Wells et al., 2009). However, the AOA are also abundant in terrestrial ecosystems with high NH_3_ concentrations (Verhamme et al., 2011). This diversity might suggest that the physiological breadth of the AOA has yet to be fully uncovered, and that the notion of the AOA as oligotrophic K-strategists might be challenged through isolation of organisms from high NH_3_ environments. On the other hand, several studies have demonstrated metabolic diversity of the terrestrial AOA (i.e., mixotrophy; Mußmann et al., 2011), and have proposed that although the abundance of the AOA is high, their contribution to ammonia oxidation is perhaps minimal. Currently, MicroTrait-N is only capable of representing organisms growing autotrophically, and does not represent the abundance of organisms with alternative metabolisms. Therefore, if an appreciable proportion of the AOA community at neutral pH is not actively oxidizing ammonia, they will not be predicted in the current model structure. Further studies into the physiology of the AOA will likely yield data that should help to constrain the models.

### Geochemical gradient simulations

MicroTrait-N attempts to predict trends in community diversity across gradients in substrate concentration, pH, and temperature.

#### pH impacts

Few studies offer an experimental analog to the simulations presented here, however, Nicol et al. (2008) examined AOA and AOB dynamics along a pH gradient (7.5–4.9) in an agricultural soil. The results of that study did not necessarily support predictions from our simulations (e.g., the AOA were observed to be the numerically dominant nitrifiers across neutral to acidic conditions), however several similarities occurred. Quantification of transcript abundance found the AOA:AOB ratio decreased with increasing pH, suggesting that the relative importance of the AOB to ammonia oxidation increases with increasing pH. Furthermore, Nicol et al. (2008) also noted the taxonomic diversity of AOB to decrease with decreasing pH. This relationship was mainly attributable to the loss of most of the *Nitrosomonas* species and several of the *Nitrosospira* clusters. Additionally, at pH ≤ 5.0 the *Nitrosospira* were the dominant bacterial nitrifying group. Our simulations reproduced some of these observations, including a drop in bacterial diversity and an increasing prominence of the AOB(7) guild (for which kinetic parameters were derived from the *Nitrosospira*) with decreasing pH.

The dominance of the AOA guild at low pH is supported by several studies (Nicol et al., 2008; Gubry-Rangin et al., 2010). However, there is also evidence of the AOA dominating nitrifier groups across a range of pH (from 8.7 to 3.5; Gubry-Rangin et al., 2011). It is not clear if this dominance is due to a physiological adaptation to low pH or to substrate availability. Nitrification rates have previously been shown to be high at low pH where rates of mineralization (and hence substrate availability) are high (Booth et al., 2005), however, (Gubry-Rangin et al., 2011) did not explicitly measure substrate concentrations in their study.

#### Temperature impacts

MicroTrait-N also simulates the relationship between temperature and the kinetics of the ammonia-monoxygenase enzyme, which purportedly has a stronger effect on the ammonia oxidation rate than substrate availability (Groeneweg et al., 1994). The MicroTrait-N relationship between temperature and activity (ammonia oxidation) was based on a previously published square-root relationship for the growth rate of bacteria (Ratkowsky et al., 1983, 2005). In the present model, nitrifier diversity and activity was highest at 25°C while the rate of N_2_O production tracked the rate of ammonia oxidation. Several laboratory and field experiments have recorded a significant positive relationship between temperature and the activity of nitrifiers (Stark, 1996; Jiang and Bakken, 1999; Avrahami and Bohannan, 2007; Bouskill et al., 2011) with a few studies noting that the relationship continues up to and above 30°C (Stark and Firestone, 1996). Understanding the relationship between temperature and nitrification is crucial to predicting future N_2_O effluxes (Avrahami and Bohannan, 2009) and future simulations should account for complex interactions between temperature, substrate, and soil moisture, all of which play a significant role in N_2_O fluxes (Avrahami and Bohannan, 2009).

#### Decoupling nitrification reactions

N_2_O is a long-lived greenhouse gas and stratospheric ozone depleting substance (Bange, 2008). The atmospheric mixing ratio of N_2_O has increased 20% since 1750 (MacFarling Meure et al., 2006) with terrestrial ecosystems the principle sources of N_2_O emissions (Pérez et al., 2001). The annual contribution of nitrification to the global N_2_O budget is currently unknown, however, in previous models the ratio of N_2_O formed to NH_3_ oxidized is generally about 0.1% (Frame and Casciotti, 2010). This relationship does not account for differences in the pathways of N_2_O production via nitrification (Frame and Casciotti, 2010).

In the current model, we simulated N_2_O production via NO_2_ detoxification and hydroxylamine decomposition. The maximal rate of N_2_O production was recorded under NO_2_ detoxification, and was approximately 150 times higher than it had been directly before NOB removal and seven times higher than the N_2_O production rate when NO_2_ did not accumulate (i.e., NOB were present and N_2_O was produced by hydroxylamine decomposition). This result might suggest that NO_2_ detoxification substantially increased N_2_O production by ammonia oxidizers upon uncoupling of the nitrification reactions. However, the toxic effect of NO_2_ reduces AOO biomass to the point where the populations crash and NH_3_ oxidation declines. This biomass change is reflected in the cumulative N_2_O production data over the 6 month simulation, which is approximately 5 times lower than that formed during full nitrification (i.e., hydroxylamine decomposition).

These model predictions are supported by previous experimental work. For example, Graham et al. (2007) observed evidence of chaotic instability in the AOB-NOB relationship resulting in significant accumulation of NO_2_ in a chemostat experiment. Furthermore, Frame and Casciotti (2010) examined pathways of N_2_O production in the marine ammonia oxidizer, *Nitrosomonas marina*. They found that the presence of excess NO_2_ in the growth medium increased N_2_O yields by an average of 70–87%, while stable isotope and ^15^N-site preference measurements determined that nitrifier-denitrification (analogous to our detoxification pathway) was responsible for the majority of N_2_O production at low oxygen (Frame and Casciotti, 2010).

#### Comparison with environmental data

We also tested our model against site-collected data from a recent study in a high-latitude site (Petersen et al., 2012). Petersen et al. (2012) sampled five-plant communities characteristic of interior Alaska, and measured the abundance of functional genes affiliated with nitrification (i.e., bacterial and archaeal ammonia monooxygenase) and potential nitrification rates. The sites were characterized by high ammonium concentrations (0.2–2.9 g m^−2^) and low pH (4.8–4.3). These sites therefore present a contrast to the earlier pH gradient analysis under a lower substrate concentration. In our pH gradient simulation the AOA dominated the low pH possibly due to low substrate availability. Conversely, at higher substrate concentrations Petersen et al. (2012) found AOB to be the dominant nitrifier in these Alaskan soil plots and the AOB *amoA* gene abundance best explained observed nitrification rates. The AOA were only minor components of the AOO communities. Recreating the initial conditions from data collected in Alaska (Carney et al., 2007; Petersen et al., 2012), we resolved plausible trends in both relative community composition (i.e., AOB biomass was higher than that of the AOA) and NH_3_ oxidation rates. Predicted NH_3_ oxidation rates correlated with NH_3_ concentrations. That the AOB dominated these communities over the AOA supports the earlier data suggesting AOO community composition is largely determined by substrate concentrations. N_2_O production generally tracked NH_3_ oxidation, indicating that N_2_O was predominantly produced via hydroxylamine decomposition. The exception was at the Bog Birch site where predicted N_2_O production was higher than a rate consistent with hydroxylamine decomposition. This result is significant given predictions of higher N_2_O production in high-latitude ecosystems dependent on N-availability (Elberling et al., 2010) and further work is warranted to understand these MicroTrait-N predictions.

In addition to replicating field studies, a major objective of any modeling approach is to test existing hypotheses. For example, our mechanistic model may be used to test existing ecological theory of the controls on ecosystem processes (in this case nitrification). At the present time, two competing hypotheses describe the relationship between community structure and ecosystem processes: The “diversity” hypothesis and the “mass-ratio” hypothesis (Grime, 1998; Green et al., 2008; Laughlin, 2011).

The “diversity hypothesis” postulates that the richness of functional groups determines the rate of ecosystem processes by a complementary association between different functional groups (e.g., Tilman et al., 1996; Laughlin, 2011). On the other hand, the “mass-ratio” hypothesis proposes that ecosystem processes are controlled by the relative abundance of different functional groups.

Our results show that these two hypotheses are both valid but at different stages of the evolving nitrifier ecosystem. Organisms achieving maximal fitness under the initial conditions can rapidly increase their biomass to dominate the nitrification process. Other guilds decline sometimes to extinction. These dynamics seemingly lend support to the “mass-ratio” hypothesis. However, as conditions change (i.e., as substrate concentrations fall), the diversity of the community becomes more important, as guilds more suited to the new conditions become numerically prominent and dominate nitrification. At the present time, we are unaware of any field studies in microbial ecology that exclusively test these theories *in situ*. The functional diversity of microbial communities, and redundancy in those communities, in addition to limitations in current methods limitations, make it difficult to attribute activity to specific groups. These limitations might be overcome in future through continued development of isotope labeling and spectroscopy methods (Hall et al., 2010) and transcriptomics (Moran et al., 2012).

## Conclusion

Trait-based microbial ecology can potentially link the observations of experimental environmental microbiology, theoretical energy, and mass exchange considerations, and quantitative modeling with an emphasis on depicting microbial diversity across spatial and temporal scales. Previous applications of the microbial trait-based approach have been successful in predicting rates of primary productivity (Follows et al., 2007), heterotrophic activity (Hall et al., 2008), and litter decomposition (Allison, 2012). We demonstrate here that trait-based representation of nitrifiers can be used to connect community diversity with activity, improve understanding of environmental controls on NH_3_ oxidation, and test hypotheses centered around the ecology of NH_3_-oxidizers and N_2_O production, issues that temporal and financial restrictions on field studies are often unable to address. An important avenue for future research is to focus on whether the integration of these microbiological diversity modules into ecosystem models can improve site, regional and global predictions of carbon and nutrient cycling.

## Conflict of Interest Statement

The authors declare that the research was conducted in the absence of any commercial or financial relationships that could be construed as a potential conflict of interest.

## Figures and Tables

**Figure A2:**
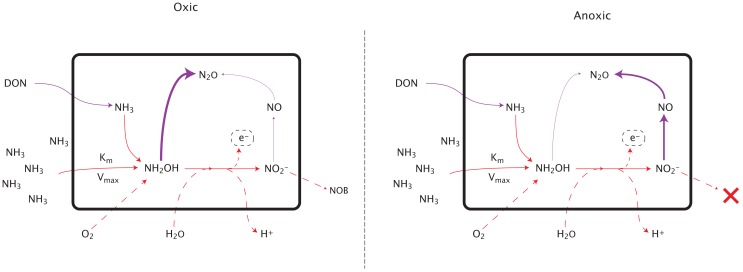
**Pathways of nitrous oxide production during nitrification**. See text for detailed explanation.

**Figure A3:**
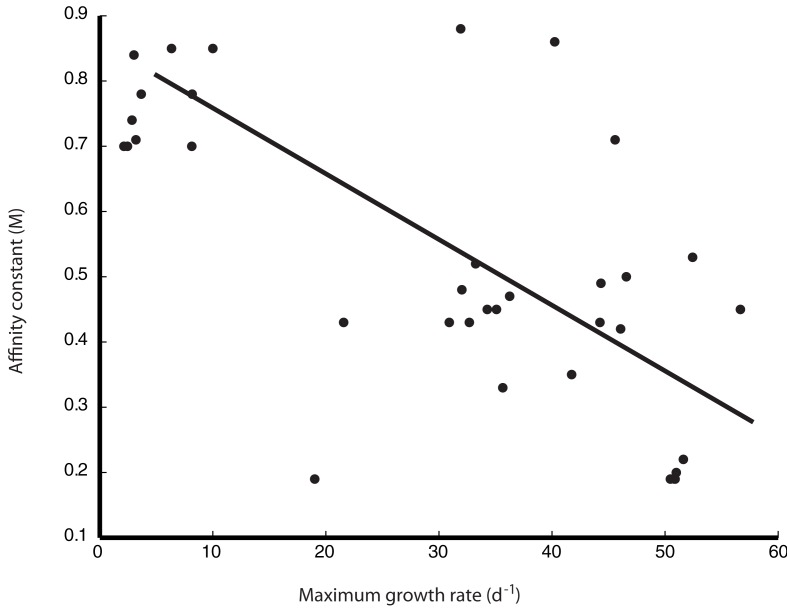
**Explicit relationship between trait parameters *K_M_* and μ_max_**.

**Figure A4:**
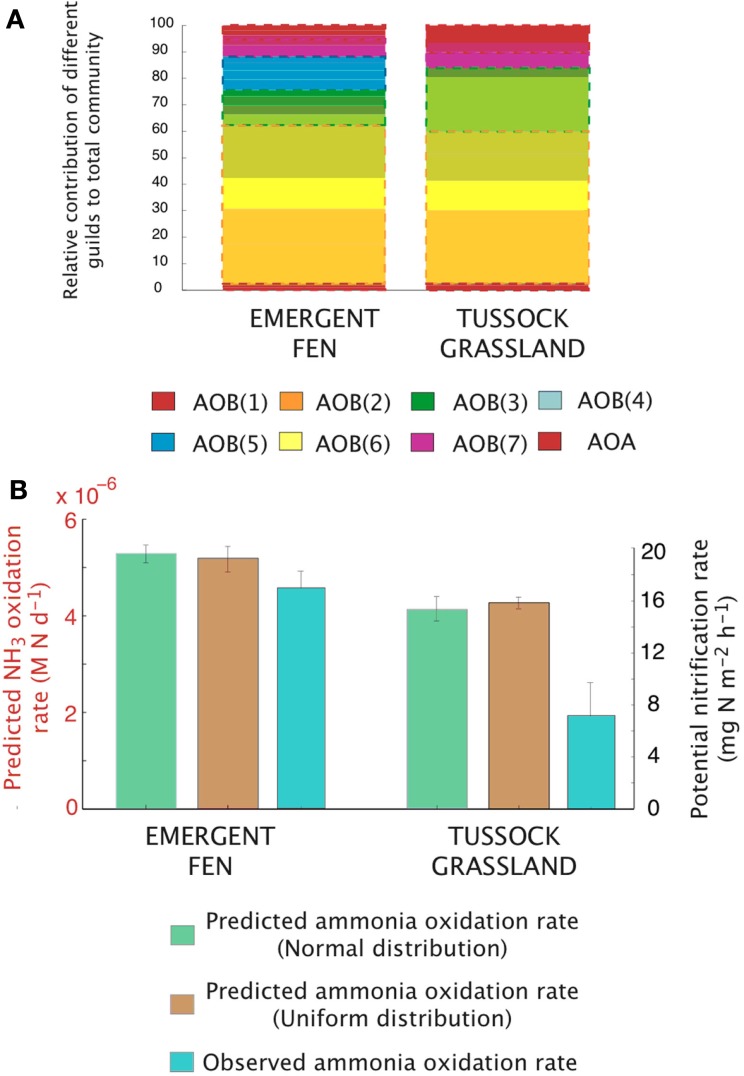
**Simulations of the activity and diversity of AOB communities in high-latitude ecosystems**. **(A)** Simulations of multiple AOB analogs (*n* = 5 analogs per guild) across the different sites. These simulations are based on a normalized distribution of trait values. Each guild is represented by a distinct color. Subtle differences in the shade of that color demarcate the different analogs/guild. A box outlines the boundaries of each guild’s biomass. Evenness statistic given above the bar plots. **(B)** Experimental observations reproduced from Petersen et al. (2012), showing the trends in potential nitrification rates under a normal distribution, a uniform distribution, and the observed NH_3_ oxidation rates.

## Appendix

### Materials and methods

#### Derivation of trait values

Numerical values for five different traits [K_M_(NH_3_), K_M_(O_2_), V_MAX_(NH_3_), μmax, R_C:N_] were taken from ecophysiological studies following an extensive literature review (Loveless and Painter, 1968; Suzuki, 1974; Suzuki et al., 1974; Drozd, 1976; Glover, 1985; Belser and Schmidt, 1979; Keen and Prosser, 1987; Prosser, 1989; Nishio and Fujimoto, 1990; Verhagen and Laanbroek, 1991; Laanbroek and Gerards, 1993; Jiang and Bakken, 1999; Schramm et al., 1999; Gieseke et al., 2001; Koops and Pommerening Röser, 2001; Cébron et al., 2003; Martens-Habbena et al., 2009; Schreiber et al., 2009). Where possible the traits were derived from the same study, however, efforts were made to ensure that the similar methodologies were used to calculate trait values (e.g., under similar pH and temperature). The different ecophysiological traits were measured in batch cultures of strains of *Nitrosomonas, Nitrosospira*, *Nitrosopumilus*, *Nitrososphaera* and *Nitrosotalea*.

– K_M_(NH_3_)/K_M_(O_2_)/V_MAX_: Enzyme kinetics (e.g., affinity constant and uptake) were calculated under substrate saturation conditions (see: Loveless and Painter, 1968; Suzuki et al., 1974; Drozd, 1976; Martens-Habbena et al., 2009). Affinity constants have previously been measured in whole cells as well as cell extracts and oxygen concentrations measured using oxygen electrodes (Suzuki et al., 1974). Enzyme uptake can be calculated using ammonia microprofiles and fitting to the Michaelis–Menton equation (e.g., Schramm et al., 1999). In the case of the AOA, *Nitrosopumilus maritimus*, affinity constants were derived using oxygen microsensors (Martens-Habbena et al., 2009), from multiple oxygen traces. Maximum uptake rate was also calculated under substrate saturation. In general, media with defined ammonia concentrations were sub-sampled over time and substrate concentrations determined fluorometrically. Uptake rates were calculated from oxygen profiles and fitted to a Michaelis Menton equation (Martens-Habbena et al., 2009).
– μmax: Maximum specific growth rate was generally estimated by measuring the evolution of NO_2_ as a proxy for growth (e.g., Loveless and Painter, 1968; Keen and Prosser, 1987). NO_2_ increases exponentially during growth and the slope of a semi-logarithmic plot of product evolution against substrate concentration is equivalent to specific growth rate.
– *R*_C:N_: The carbon yield from nitrification was determined in continuous or chemostat cultures (e.g., Belser, 1979; Belser and Schmidt, 1979; Glover, 1985; Keen and Prosser, 1987) by measuring cell number (e.g., using a spectrometric bacterial counter) and the production (AOB), or draw down (NOB), of NO_2_.

**Table A1:** **Initial inputs for model simulation of the Petersen dataset**.

Plant community type	pH	NH_3_ (g m^3^)	Potential nitrification rate	16s bacterial: archaea
Black spruce	4.8	0.2	2	15
Black bog	4.3	0.2	1	37.5
Emergent fen	4.5	2.9	18	10
Rich fen	4.7	1.1	5	3
Tussock grassland	4.7	1.5	7	10
